# The genome sequence of *Saccopteryx leptura, Schreber, 1774 *(Chiroptera, Emballonuridae, Saccopteryx)

**DOI:** 10.12688/wellcomeopenres.25254.1

**Published:** 2026-02-05

**Authors:** Ine Alvarez van Tussenbroek, Mirjam Knörnschild, Martina Nagy, Martin Pippel, Thomas Brown, Sylke Winkler, Myrtani Pieri, Meike Mai, Eugene W Myers, Emma C Teeling, Sonja Vernes, Bat1K Consortium

**Affiliations:** 1 School of Biology, University of St Andrews, St Andrews, UK; 2 Institute of Biology, Leiden University, 2300, RA Leiden, PO Box 9505, The Netherlands; 3 Museum für Naturkunde, Leibniz-Institute for Evolution and Biodiversity Science, Berlin, Germany; 4 Smithsonian Tropical Research Institute, Balboa Ancon, Panama City, Panama; 5 Evolutionary Ethology Lab, Humboldt-Universität zu Berlin, Berlin, Germany; 6 Max Planck Institute of Molecular Cell Biology and Genetics, Pfotenhauerstr. 108, 01307, Dresden, Germany; 7 Center for Systems Biology, Dresden, Pfotenhauerstr. 108, 01307, Dresden, Germany; 8 DRESDEN concept Genome Center, Center for Molecular and Cellular Bioengineering, Technische Universität, Dresden, 01307, Dresden, Germany; 9 Department of Life Sciences, School of Life and Health Sciences, University of Nicosia, Nicosia, Cyprus; 10 School of Biology and Environmental Science, University College Dublin,, Dublin, Ireland; 11 Wellcome Sanger Institute, Wellcome Genome Campus, Cambridgeshire, CB10 1SA, UK

**Keywords:** Saccopteryx leptura, genome sequence, chromosomal, Bat1K

## Abstract

We present a genome assembly from an individual male *Saccopteryx leptura* (Chordata; Mammalia; Chiroptera; Emballonuridae). The genome sequence is 2.6 Gb in span. Most of the assembly is scaffolded into 14 chromosomal pseudomolecules, with the X chromosome assembled. Due to the Y chromosome being very short in this species, assembly has not yet been successful.

## Species taxonomy

Eukaryota; Metazoa; Chordata; Craniata; Vertebrata; Euteleostomi; Mammalia; Eutheria; Laurasiatheria; Chiroptera; Yangochiroptera; Emballonuroidea; Emballonuridae; Emballonurinae; *Saccopteryx*; *Saccopteryx leptura* ( Grau *et al.*, 2017; Meredith *et al.*, 2011; Schreber, 1774; Teeling *et al.*, 2005).

## Introduction

Bats from the family Emballonuridae (or sheath-tailed bats) are aerial insectivores. They are found in Africa and Indo-Malayan, Australian, Neotropical, and Holartic regions (Mammals of the world - Bats page334). Although typically found in tropical forest regions, a few species have been found in semiarid and desert regions (Mammals of the world - Bats page338).

**Figure 1.**
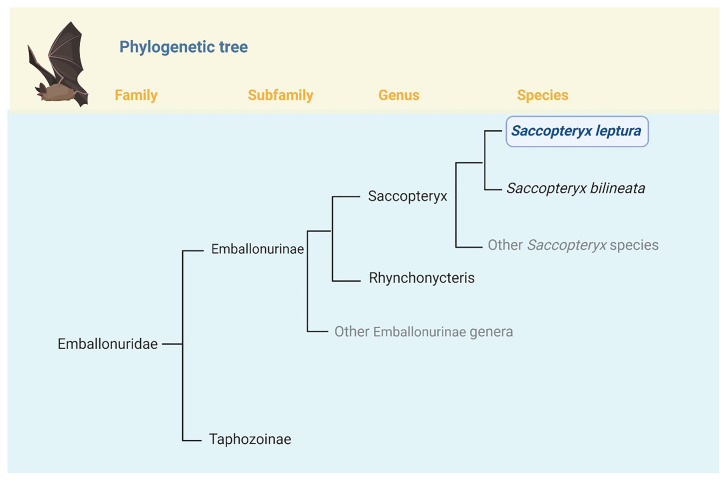
Position of *Saccopteryx leptura* in the phylogeny of Family Emballonuridae. The bat *Saccopteryx leptura* is one of the five species currently recognized in the genus Saccopteryx ( Illiger, 1811) *. Saccopteryx leptura* belongs to the Subfamily Emballonurinae, which currently includes twelve genera and thirty-seven species ( Bucknell University, n.d.; Integrated Taxonomic Information System, 2025) *Saccopteryx leptura* is a monotypic species.

The Emballonuridae family comprises two subfamilies: Taphozoinae and Emballonurinae. Emballonurinae consists of 11 genera and 33 species ( Simmons & Cirranelo, 2025). The genus *Saccopteryx* is within Emballonurinae and comprises five species: *Saccopteryx leptura, S. bilineata, S. canescens, S. gymnura and S. antioquensis. S. leptura* is a monotypic species as shown in Figure 1.

*Saccopteryx leptura,* the lesser sac-winged bat or lesser white-lined bat, has been found in tropical regions in middle and south America from the south of Mexico, Belize and Guatemala to the north of Bolivia and center of Brazil (Mammals of the world, Bats page 372; Yancey *et al.*, 1998). They are found in lowland regions, in forested areas, often below 100m although they sometimes are located up to 914m above sea level (Mammals of the world, Bats page 372).

**Figure 2.**
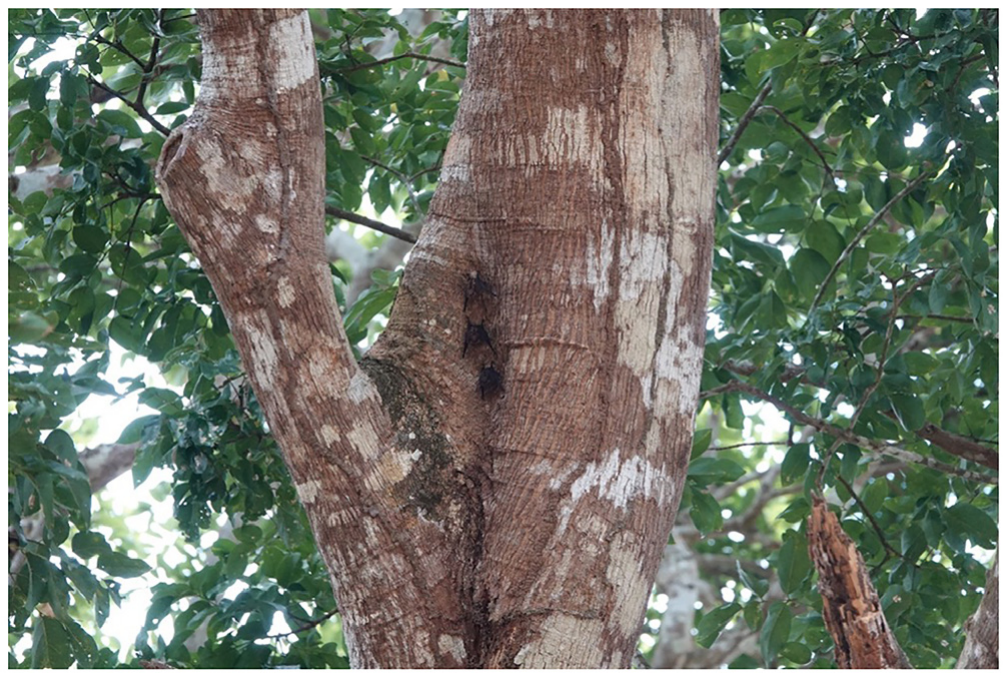
Lesser sac-winged bats, *Saccopteryx leptura*. Individuals of *Saccopteryx leptura*. This species can be found often on trees. These individuals were found several meters above ground on a tree. They have brown fur with two light stripes on the back. Photos taken in Gamboa, Panama by Ine Alvarez van Tussenbroek.

The lesser sac-winged bats are small bats, 38–51mm from the head to the end of the body with added 9–19mm of tail length, the forearm length is 37–43mm and they weigh around 3–6g (Mammals of the world - Bats page 372). These bats are brown with two light-colored wavy lines on the back. *Saccopteryx leptura* has a similar appearance to the sister species *Saccopteryx bilineata* although it can be differentiated by its lighter fur color and smaller size ( Figure 2). *Saccopteryx leptura* is classified in the IUCN Red List as a species of Least Concern (lc).

*Saccopteryx leptura* hunts aerial insects using echolocation in edge spaces. *Saccopteryx leptura* emits short multiharmonic echolocation calls through the mouth with the second harmonic containing most of the call’s energy. Echolocation calls consist of a central, shallow modulated narrowband (QCF, quasi constant frequency) part, with two short FM (frequency modulated) sweeps at the beginning and the end of the call. This bat produces search calls of alternating frequencies. The peak frequency of the QCF part of the lower echolocation call averages 51.3 (±1.8) and the higher call has an average peak frequency of 54.6 (±1.8) kHz in Central America ( Jung *et al.*, 2007).

## Genome sequence report

**Figure 3.**
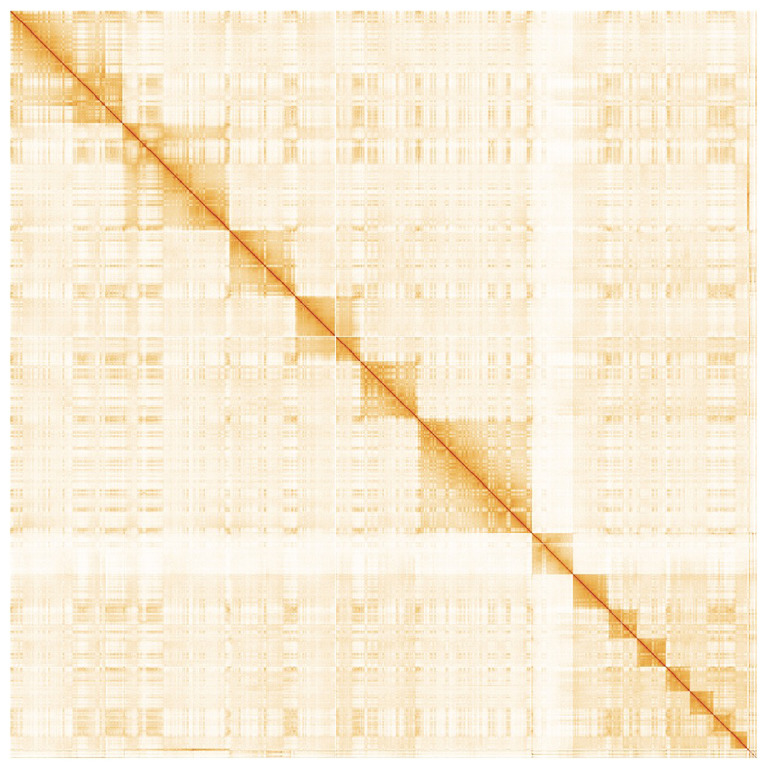
Hi-C Contact Map of the *Saccopteryx leptura* assembly with *14* chromosomes, visualized using HiGlass.

**Figure 4.**
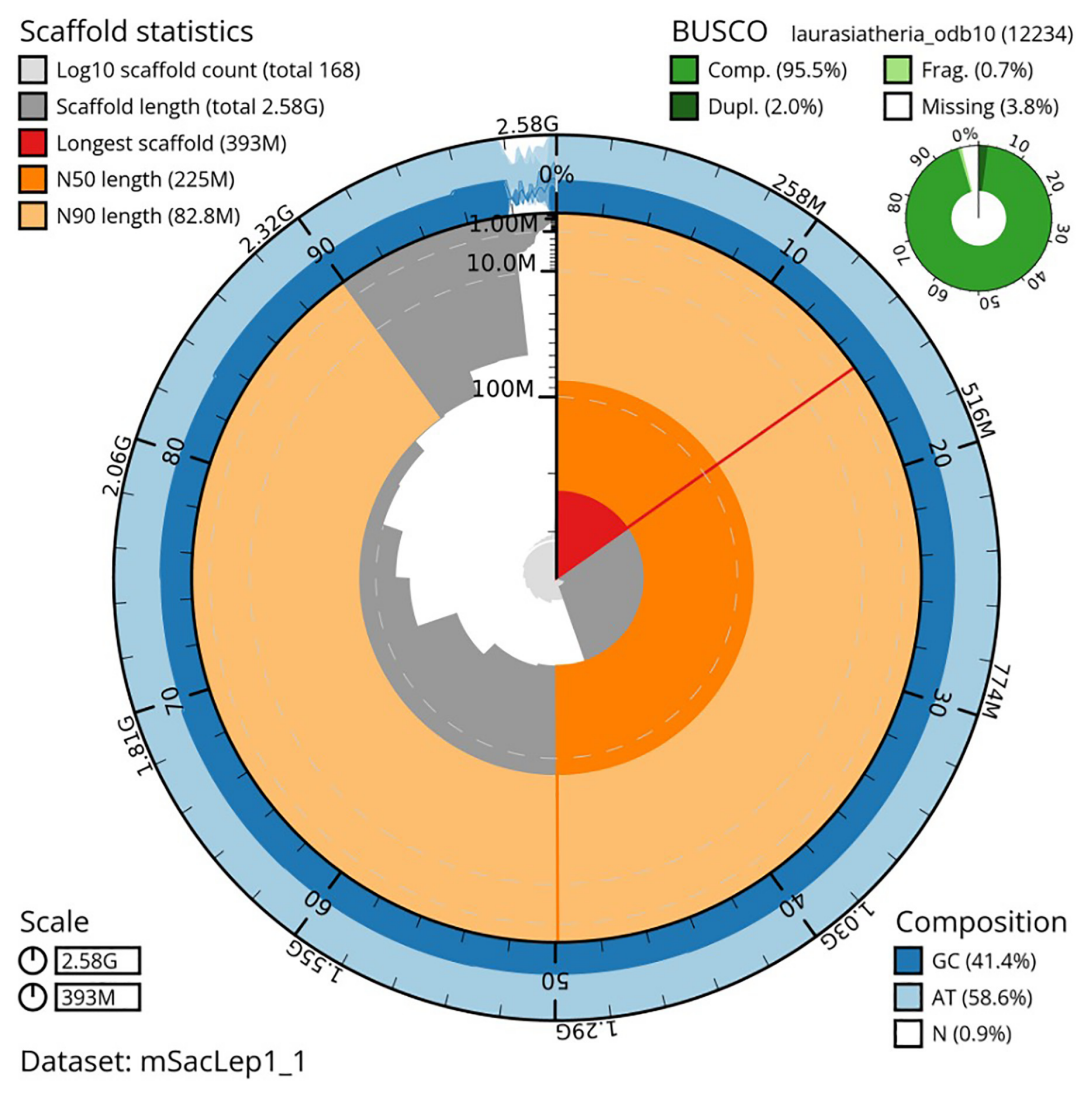
Genome assembly metrics generated using blobtoolkit for the *Saccopteryx leptura* genome assembly. The larger snail plot depicts scaffold statistics including N50 length (bright orange) and base composition (blue). The smaller plot shows BUSCO completeness in green.

The genome was sequenced from a single male *Saccopteryx leptura* collected on March 23rd, 2019, from a tree in Gamboa, Panama (GPS coordinates: 9.119726, -79.702898). A total of 69-fold coverage in Pacific Biosciences CLR long reads was generated, with read N50 of 30kb. Primary assembly contigs were generated using Falcon and Falcon-Unzip, followed by removal of retained haplotigs with purge-dups, resulting in 752 sequence contigs and a contig N50 of 17.8Mb. The sequences were scaffolded with 10X Genomics linked-reads using Scaff10X, Bionano optical maps using Bionano-Solve and chromosome confirmation Hi-C data using Salsa2 followed by manual curation and error-correction using the PacBio CLR and 10X linked-reads. The final assembly has a total length of 2.58 Gb in 170 sequence scaffolds with a scaffold N50 of 225 Mb ( Table 1). The majority, 98%, of the assembly sequence was assigned to 14 chromosomal-level scaffolds, representing 13 autosomes (numbered by sequence length, and the X sex chromosome). Due to the Y chromosome being very short in this species (See also Baker & Jordan, 1970), assembly has not yet been successful. Chromosomal pseudomolecules in the genome assembly of *S. leptura* are shown in Table 2. Hi-C contact mapping supporting the chromosomal-scale assembly is shown in Figure 3. The assembly has a BUSCO ( Simao *et al.*, 2015) completeness of 95.4% using the laurasiatheria reference set (n=12,234). Summary assembly metrics and completeness statistics are visualised in Figure 4. While not fully phased, the assembly deposited is of one primary haplotype and a set of alternate contigs.

**Table 1.** Genome data for *Saccopteryx leptura*.

*Project accession data*	
Assembly identifier	mSacLep1_pri_phased_curated
Species	*Saccopteryx leptura*
Specimen	Liver, muscle
NCBI taxonomy ID	NCBI: txid249018
BioProject	Bat1K: Accession: PRJNA489245; ID: 489245 *Saccopteryx leptura* Accession: PRJNA1017487
BioSample ID	SAMN37390139; mSacLep1
Isolate information	Male - Liver
*Raw data accessions*	
Pacific Biosciences SEQUEL II	SRX24007513
10X Genomics Linked-reads Illumina	SRX24000462
Hi-C Illumina	SRX24000691
BioNano map	SUPPF_0000005634
Assembly accession	GCA_036850995.1
Accession of Alternative haplotype	GCA_036851165.1
Span (Mb)	2,580
Number of contigs	725
Contig N50 length (Mb)	17.8
Number of scaffolds	167
Scaffold N50 length (Mb)	224.8
Longest scaffold (Mb)	393

* BUSCO scores based on the mammalia_odb10 BUSCO set using v5.1.1. C= complete [S= single copy, D=duplicated], F=fragmented, M=missing, n=number of orthologues in comparison. * *Saccopteryx leptura* BUSCO scores based on laurasiatheria_odb10 BUSCO set v5.1.1.

**Table 2.** Chromosomal pseudomolecules in the genome assembly of *Saccopteryx leptura*. ENA accession Chromosome Size (Mb) GC%. The chromosome number of *Saccopteryx leptura* is 2n= ***28*** (See also Baker & Jordan, 1970). The Y chromosome was not assembled.

ENA accession	Chromosome	Size (Mb)	GC%
CM072134.1	1	392.7	41.63
CM072135.1	2	386.3	42.38
CM072136.1	3	372.6	41.63
CM072137.1	4	224.8	40.73
CM072138.1	5	221.1	41.73
CM072139.1	6	196.7	42.49
CM072147.1	X	138.9	39.18
CM072140.1	7	121.8	40.39
CM072141.1	8	98.21	39.92
CM072142.1	9	96.16	43.47
CM072143.1	10	82.79	43.22
CM072144.1	11	81.03	42.33
CM072145.1	12	58.07	41.61
CM072146.1	13	57.44	46.20

## Methods

The *S. leptura* specimen was a male individual collected during a field expedition in Gamboa, Panama, led by Mirjam Knörnschild. *Saccopteryx leptura* was first identified by the tree (a group of 3 *S. leptura* bats were hanging from the tree close to each other in daylight in a park). The size of the bat, the brown fur and the two light colored lines on the back of these bats determined the identification of this species as described previously ( Yancey *et al.*, 1998). The group of *S. leptura* was first identified by a student under the supervision of M. Knörnschild. The bat was caught by using high stairs and a long hand net that could reach the top of the tree. The animal was identified as *S. leptura* based on location where it was found, and morphological traits described by Yancey *et al.* (1998). After confirmation of the species and the sex it was placed in a fabric bag and taken to the laboratories at the Smithsonian Institute in Gamboa for tissue harvesting. After species identification, the individual was euthanized humanely by experienced researchers while monitoring and prioritizing the reduction of stress and suffering of the animal. The animal was euthanized by overdose of isoflurane inhalation (Formula CHF₂OCClHCF₃, CAS number 26675-46-7; Piramal Critical Care). Isoflurane inhalation is a humane, approved euthanasia method that rapidly induces unconsciousness and death. Due to the high respiration rate of bats, the individual was rendered unconscious within seconds, with death occurring within one to two minutes without observable distress. Absence of breathing and reflexes was confirmed, and isoflurane exposure was extended for an additional one minute after cessation of respiration. Death was immediately confirmed by decapitation prior to tissue collection. Capture and sampling were done under the project proposal 2019-0301-2022 approved by the Smithsonian Tropical Research Institute and the STRI Animal Care and Use Commitee (ACUC) and collection and export was conducted under the collecting field number issued by UNARGEN SC/A-3-19. All work was conducted with approval by the Panamanian Ministry of Environment (Mi Ambiente). Euthanasia was performed using isoflurane inhalation overdose by trained personnel, in accordance with approved veterinary guidelines, followed by confirmation of death prior to tissue collection. Tissues were removed from the subject individual immediately following euthanasia and were flash-frozen in liquid nitrogen and stored in a freezer at -80°C until shipping on dry ice, maintaining the cold chain. All data were recorded and reported in accordance with the ARRIVE guidelines ( Kilkenny *et al.*, 2010). See data availability section and Table 1.

### Bionano agarose plug based isolation of ultra-long genomic DNA

Ultra-long genomic DNA (gDNA) of *Saccopteryx leptura* liver tissue was extracted according to the Bionano Prep ^™^ Animal tissue DNA isolation soft tissue protocol (document number 30077, Bionano, San Diego, CA). In brief, about 35 mg of snap-frozen liver tissue was homogenized in a tissue grinder directly followed by a mild Ethanol fixation. Homogenized tissue was embedded into agarose plugs and treated with Proteinase K and RNase A. Genomic DNA was extracted from agarose plugs by agarose digestion and was finally purified by drop dialysis against 1x TE. PFGE (Pulsed-field gel electrophoresis) revealed ultra-long DNA molecule length of 100 kb up to 500 kb.

### Pacific Biosciences long insert library preparation

One long insert (continuous long read CLR) library of *Saccopteryx leptura* was prepared as recommended by Pacific Biosciences (PacBio, Menlo Park, CA) according to the ‘Guidelines for preparing size-selected 20 kb SMRTbell ^™^ templates. In summary, ultra-long gDNA was post-purified by pre-washed 1x AMPure XP beads according to the manufacturer’s guidelines. The post-purified ultra-long gDNA was sheared to 75 kb fragments with the MegaRuptor ^™^ device (Diagenode) and 7 µg sheared gDNA was used for PacBio CLR library preparation. PacBio SMRTbell ^™^ libraries were size selected for fragments larger 25 kb making use of the SAGE BluePippin ^™^ devise. This size selected PacBio CLR library was loaded with 40 pM on three SMRT cells on the SEQUEL2. SMRT sequencing was done with the PacBio SEQUEL2 binding kit 2.0 and sequencing chemistry 2.0; movie time was 15 hours for a total of three SMRT cells resulting in 344 Gbp of unique insert reads.

### Bionano optical mapping of megabase-size gDNA

Ultra-long gDNA of *Saccopteryx leptura* was labelled as described in the Bionano Prep direct label and stain (DLS) protocol (Document number 30206). The ultra-long gDNA was tagged with the nicking-free DLE enzyme. One flow cell of the labelled gDNA was run on the Bionano Saphyr instrument and circa 120X genome coverage of molecules longer than 150 kbp was achieved.

### 10x linked reads

Linked Illumina reads of *Saccopteryx leptura* were generated by using the 10x Genomics Chromium ^™^ genome application following the Genome Reagent Kit Protocol v2 (Document CG00043, Rev B, 10x Genomics, Pleasonton, CA). In brief, 1 ng of ultra-long genomic DNA was partitioned across over 1 million Gel bead-in-emulsions (GEMS) using the Chromium ^™^ devise. Single gDNA molecules were amplified in these individual GEMS in an isothermal incubation using primers that contain a specific 16 bp 10x barcode and the Illumima ^®^ R1 sequence. After breaking the emulsions, pooled amplified barcoded fragments were purified, enriched, and went into Illumina sequencing library preparation as described in the protocol. Pooled Illumina libraries were sequenced to a 90X genome coverage on an Illumina NovaSeq 6000 instrument on an S4 flow cell in XP mode with 300 cycles.

### Hi-C chromatin confirmation capture

Chromatin conformation capturing of *Saccopteryx leptura* chromatin was done making use of the ARIMA Hi-C+ Kit (Material Nr. A410110) and followed the user guide for animal tissues (ARIMA-Hi-C 2.0 kit Document Nr: A160162 v'00). In brief, circa 25 mg flash-frozen powdered muscle tissue was crosslinked chemically. The crosslinked genomic DNA was digested with the restriction enzyme cocktail consisting of four restriction enzymes. The 5’-overhangs are filled in and labelled with biotin. Spatially proximal digested DNA ends were ligated and finally the ligated biotin containing fragments were enriched and went for Illumina library preparation, which followed the ARIMA user guide for Library preparation using the Kapa Hyper Prep kit (ARIMA Document Part Number A160139 v00). The barcoded Hi-C libraries run on a NovaSeq6000 S4 flow cell in XP mode with 300 cycles to 120x genome coverage.

**Figure 5.**
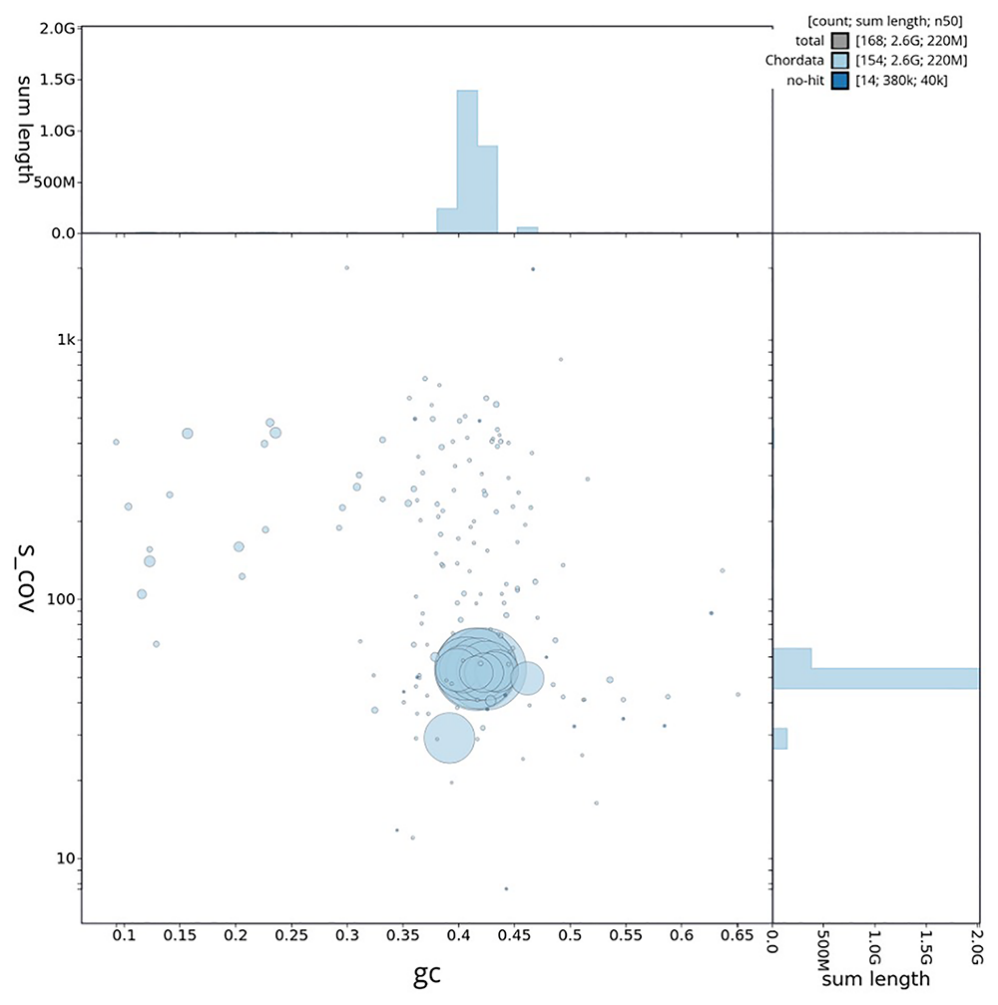
GC coverage plot generated for the *Saccopteryx leptura* assembly using blobtoolkit. Individual chromosomes and scaffolds are represented by each circle. The circles are sized in proportion to chromosome/scaffold length. Histograms show the sum length of chromosome/scaffold size along each axis. Color of circles indicate taxonomic hits of each Phylum represented in the assembly.

**Figure 6.**
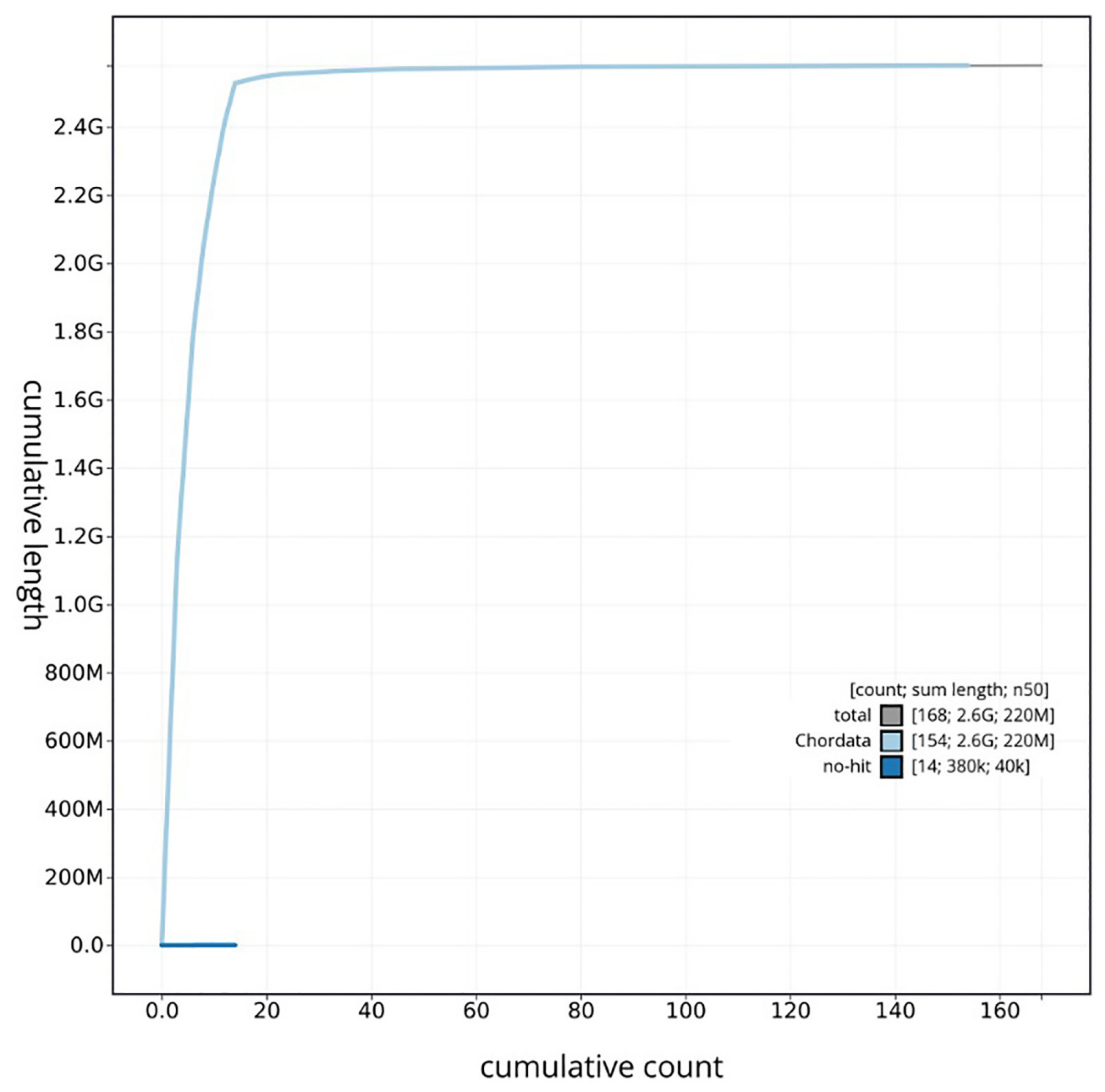
Cumulative sequence plot generated for the *Saccopteryx leptura* assembly using blobtoolkit. The grey line shows the cumulative length for all chromosomes/scaffolds in the assembly. Colored lines represent Phylum represented in the assembly.

Assembly was carried out following the Vertebrate Genome Project pipeline v1.6 ( Rhie *et al.*, 2021) as follows. The initial set of contigs were generated using Falcon and Falcon-Unzip (Falcon-kit v1.8.1). Haplotypic duplication was identified and removed with purge dups (v1.2.3) ( Guan *et al.*, 2020). The quality of the assembly was evaluated using Merqury ( Rhie *et al.*, 2020) and BUSCO ( Manni *et al.*, 2021). Scaffolding with 10X data was carried out with Scaff10X (commit bc3a0cb), Bionano data with Bionano Solve (v 3.6.1) and Hi-C data with SALSA2 (commit e6e3c77) ( Ghurye *et al.*, 2019). HiGlass ( Kerpedjiev *et al.*, 2018) was implemented to generate Hi-C contact maps and perform manual curation of scaffolds into chromosomes. Initial error-correction (polishing) and gap-closing was performed by mapping the CLR reads to the genome with pbmm2 (v1.7.0) followed by polishing using gcpp (v2.0.2). Two further rounds of polishing were implemented mapping the 10X reads to the genome using Longranger (v2.2.2), variants called using Freebayes (v1.3.2) with argument “-g 600” and then the consensus called with bcftools view (v1.12) with argument “'-i 'QUAL>1 && (GT="AA" || GT=“Aa”)'" and bcftools consensus with argument “-Hla”. Finally, chromosomes were phased by applying an adapted version of the DipAsm pipeline ( Garg *et al.*, 2020), calling heterozygous sites using 10x reads and creating phase blocks by linking heterozygous sites in 10x, Hi-C and PacBio reads using Hapcut2 (git commit 1ee1d58) and whatshap (v1.6). The whatshap-tagged PacBio and 10x reads, binned into H0 and H1, and H0 and H2, were then used to haplotype-polish the chromosomes. Arrow was run using the combined H0 and H1 haplotagged PacBio CLR reads, followed by freebayes polishing as above using the combined H0 and H1 haplotagged 10x reads as above. Correspondingly the H2 and H0 tagged reads were used for the second haplotype. Figure 4– Figure 6 were generated using BlobToolKit ( Challis *et al.*, 2020). Software utilised for *S. leptura* analysis is depicted in Table 3. GC coverage and taxonomic assignment across scaffolds are shown in Figure 5. Cumulative scaffold length and taxonomic representation across the assembly are illustrated in Figure 6.

**Table 3.** Software tools used.

Software tool	Version	Source
bamUtil	1.0.15	https://genome.sph.umich.edu/wiki/BamUtil:_bam2FastQ
FastQC	0.11.9	https://www.bioinformatics.babraham.ac.uk/projects/fastqc/
MultiQC	1.13	https://github.com/ewels/MultiQC
Genomescope	2.0	https://github.com/tbenavi1/genomescope2.0
Falcon-kit	1.8.1	https://github.com/PacificBiosciences/FALCON
purge_dups	1.2.3	https://github.com/dfguan/purge_dups
Longranger	2.2.2	https://support.10xgenomics.com/genome-exome/software/downloads/latest
Scaff10X	4.1	https://github.com/wtsi-hpag/Scaff10X
Bionano Solve	3.5.1	https://bionano.com/software-downloads/
BUSCO	5.1.1	https://busco.ezlab.org/
Merqury	1.3	https://github.com/marbl/merqury
Assembly-stats	17.02	https://github.com/rjchallis/assembly-stats
Arima-HiC Mapping Pipeline	-	https://github.com/ArimaGenomics/mapping_pipeline
SALSA	2.2	https://github.com/marbl/SALSA
hiGlass	1.11.7	https://github.com/higlass/higlass
samtools	1.9	https://www.htslib.org/
BlobToolKit	3.2.7	https://github.com/blobtoolkit/blobtoolkit
Freebayes	1.3.2	https://github.com/freebayes/freebayes
Bcftools	1.12	https://github.com/samtools/bcftools
pbmm2	1.7.0	https://github.com/PacificBiosciences/pbmm2
Gcpp	2.0.2	https://github.com/PacificBiosciences/gcpp
hapcut2	git commit 1ee1d58	https://github.com/vibansal/hapcut2
whatshap	1.6	https://github.com/whatshap/whatshap

## Ethics

Capture and sampling were done under the project proposal 2019-0301-2022 approved by the Smithsonian Tropical Research Institute and the STRI Animal Care and Use Committee (ACUC) and collection and export was conducted under the collecting field number issued by UNARGEN SC/A-3-19. All work was conducted with approval by the Panamanian Ministry of Environment (Mi Ambiente). Euthanasia was performed using isoflurane inhalation overdose in accordance with approved veterinary guidelines. All efforts were made to ameliorate suffering of animals.

## Data Availability

The *Saccopteryx leptura* genome sequencing initiative is part of the Bat1K genome sequencing project. The genome assembly is released openly for reuse. Data is available from public repositories as per Table 1. The primary genome assembly can be found in the European Nucleotide Archive: *Saccopteryx leptura.* Accession number: GCA_036850995.1 https://www.ncbi.nlm.nih.gov/datasets/genome/GCA_036850995.1/ NCBI BioProject: PRJNA1017170, Saccopteryx leptura principal haplotype genome sequencing. Isolate: mSacLep1 under the Bat1K BioProject PRJNA489245. Zenodo. The genome sequence of Saccopteryx leptura, Schreber, 1774 (Chiroptera, Emballonuridae, Saccopteryx). https://doi.org/10.5281/zenodo.18268510. ( bat1k, 2026) This project contains the following underlying data: ARRIVE Checklist Data is available under the terms of the Creative Commons Attribution 4.0 International.
