# Supplementary material for: The genome sequence of Saccopteryx leptura, Schreber, 1774 (Chiroptera, Emballonuridae, Saccopteryx)
Source: Wellcome Open Res. 2026 Feb 5;11:92. [Version 1] doi: 10.12688/wellcomeopenres.25254.1 (PMC13047367; doi:10.12688/wellcomeopenres.25254.1)
Supplement: Peer review history

# Reviewer report: Reviewer response for version 1

**DOI:** 10.21956/wellcomeopenres.27833.r150661

**Published:** 2026-04-02

**Authors:** Guilherme Siniciato Terra Garbino

**Affiliations:** 1 Federal University of Vicosa, Viçosa, State of Minas Gerais, Brazil

This is an interesting article on the genome sequence of Sacopteryx leptura. I have made a few specific comments below:

 In the "species taxonomy" state also that it belongs to tribe Diclidurini

 Second pargraph of the Intro, please change " *S. leptura* is a monotypic species as shown in Figure 1." to " *Saccopteryx leptura* is a monotypic species as shown in Figure 1.", to avoid beginning a sentence with an abbreviation.

 Figure 1 legend, please italicize "Saccopteryx" in "in the genus Saccopteryx"

 Here " to the north of Bolivia and center of Brazil" - it actually reaches southeastern Brazil (https://checklist.pensoft.net/article/18211/)

 Please remove the "The" here: "The lesser sac-winged bats are small bats"

Are sufficient details of methods and materials provided to allow replication by others?

Yes

Is the rationale for creating the dataset(s) clearly described?

Yes

Are the datasets clearly presented in a useable and accessible format?

Yes

Are the protocols appropriate and is the work technically sound?

Yes

Reviewer Expertise:

Systematics, taxonomy and biogeography of Neotropical mammals

I confirm that I have read this submission and believe that I have an appropriate level of expertise to confirm that it is of an acceptable scientific standard.

# Reviewer report: Reviewer response for version 1

**DOI:** 10.21956/wellcomeopenres.27833.r147064

**Published:** 2026-03-14

**Authors:** Michael R Buchalski, Samantha Capel

**Affiliations:** 1 California Department of Fish and Wildlife, Sacramento, California, USA; 2 California Department of Fish and Wildlife, Sacramento, Sacramento, California, USA

This Data Note describes the methodology and results of generating a genome assembly for *Saccopteryx leptura*. They describe the laboratory methodologies for sample collection, generating and sequencing DNA libraries, and assembly as well as where to find the generated assembly and associated datasets.

 There is no justification given for generating this genome assembly. A paragraph in the introduction indicating this as the first genome assembly for the species along with the importance of generating reference genomes, particularly within the framework of the Bat1K initiative, would suffice. Otherwise, the authors are thorough in their description of the methods, which are well in line with the current expectations for genome assembly methods. The results are well summarized in table and figure form and the locations for data availability are sufficiently detailed.

Are sufficient details of methods and materials provided to allow replication by others?

Yes

Is the rationale for creating the dataset(s) clearly described?

No

Are the datasets clearly presented in a useable and accessible format?

Yes

Are the protocols appropriate and is the work technically sound?

Yes

Reviewer Expertise:

Population genomics, genome assembly, conservation genomics

We confirm that we have read this submission and believe that we have an appropriate level of expertise to confirm that it is of an acceptable scientific standard.
